# O18Quant: A Semiautomatic Strategy for Quantitative Analysis of High-Resolution ^16^O/^18^O Labeled Data

**DOI:** 10.1155/2014/971857

**Published:** 2014-05-11

**Authors:** Yan Guo, Masaru Miyagi, Rong Zeng, Quanhu Sheng

**Affiliations:** ^1^Center for Quantitative Sciences, Vanderbilt University, Nashville, TN 37027, USA; ^2^Center for Proteomics and Bioinformatics, School of Medicine, Case Western Reserve University, Cleveland, OH 44106, USA; ^3^Key Laboratory of Systems Biology, Institute of Biochemistry and Cell Biology, Shanghai Institutes for Biological Science, Chinese Academy of Sciences, Shanghai 200031, China

## Abstract

Proteolytic ^18^O-labeling has been widely used in quantitative proteomics since it can uniformly label all peptides from different kinds of proteins. There have been multiple algorithms and tools developed over the last few years to analyze high-resolution proteolytic ^16^O/^18^O labeled mass spectra. We have developed a software package, O18Quant, which addresses two major issues in the previously developed algorithms. First, O18Quant uses a robust linear model (RLM) for peptide-to-protein ratio estimation. RLM can minimize the effect of outliers instead of iteratively removing them which is a common practice in other approaches. Second, the existing algorithms lack applicable implementation. We address this by implementing O18Quant using C# under Microsoft.net framework and R. O18Quant automatically calculates the peptide/protein relative ratio and provides a friendly graphical user interface (GUI) which allows the user to manually validate the quantification results at scan, peptide, and protein levels. The intuitive GUI of O18Quant can greatly enhance the user's visualization and understanding of the data analysis. O18Quant can be downloaded for free as part of the software suite ProteomicsTools.

## 1. Introduction

Proteomic research refers to high-throughput studies of large amount of proteins. With the rise of high-throughput sequencing, many researchers have shifted their focus to the genome using RNAseq technology. However, high-throughput sequencing technology does not help us answer proteomic questions, and the study of proteomics provides an entirely different level of genomic understanding. For example, messenger RNA abundance does not always translate into protein abundance [1], posttranslational modification is not observable through RNAseq, and protein degradation rate may play a significant role in protein content [2]. Thus, proteomics should always be a pivotal part of our quest to understand the complete human biology.

Mass spectrometry is a powerful method for quantifying proteins. It produces spectra of masses of molecules from the protein. The spectra can be used to determine the isotopic signature of the sample. Labeling is a nonoptional step in mass spectrometry. There are currently four major labeling techniques: SILAC, ICAT, ITRAQ, and ^18^O. Compared to other labeling techniques, ^18^O labeling requires less reagents and synthetic steps. However, ^18^O labeling does require extra time and labels. Our software O18Quant is specially designed for ^18^O labeled data.

Isotopic labeling has been commonly used for the quantification of peptides and proteins in biological samples [3, 4]. A natural extension of isotopic labeling is isotope dilution analysis [5]. Isotope dilution analysis is usually conducted in comparative scenarios, because it is difficult to accurately obtain absolute measurement [6]. During the comparative method, usually, labeled proteins obtained from an unstressed system are pooled together with the same amount of unlabeled protein from a second stressed system. Then, mass spectrometry is performed on the combined pool to obtain differentially expressed proteins between stressed and unstressed systems.

Researchers have developed a more convenient isotope dilution approach taking advantage of ^18^O, which can be easily added to peptides by the enzyme-catalyzed incorporation of oxygen in the C-terminal carboxylic acids during the digestion step [7]. A quick equilibrium can be achieved by exchanging at either or both of the C-terminal carboxyl oxygen atoms if the kinetics for complex formation is faster than the digestion time. Thus, the ^18^O/^16^O ratio can be used to estimate the relative abundance of the protein between the stressed and unstressed systems. Since the early 2000s, proteolytic ^18^O-labeling has been commonly adopted for use in comparative proteomics because it can uniformly label all peptides from different kinds of proteins [8–10].

During the last ten years, multiple algorithms [11–15] have been developed to analyze high-resolution proteolytic ^16^O/^18^O labeled mass spectra. Unfortunately, the majority of these algorithms lack actual implementation. Few software packages are freely available for users. Thus, there is a strong interest in developing a software package for ^16^O/^18^O labeled protein ratio calculation and validation. Here we present a semiautomatic tool, O18Quant, for analysis of such data. O18Quant differs from other previously published algorithms in two major ways. First, O18Quant has been implemented using C# and R, and a useable package is available for download. Second, O18Quant uses RLM to compute protein ratios. RLM accounts for the effect of outlier peptides instead of completely removing them iteratively. RLM has also been used in the evaluation of peptide identifications [16], reducing technical variability in functional protein microarrays [17], and SILAC peptide ratio calculation [18].

O18Quant calculates the protein ratio automatically based on user-predefined parameters such as purity of ^18^O water and resolution of mass spectrometry. Then the quantification results can be manually validated at scan, peptide, and protein levels through a user-friendly GUI. Only protein quantifications that pass quality control at all three levels are considered to be used in further analysis. O18Quant and its source code can be downloaded freely from https://github.com/shengqh/RCPA.Tools/releases/ and its detailed introduction can be viewed at https://github.com/shengqh/RCPA.Tools/wiki/.

## 2. Materials and Methods

### 2.1. Preparation of ^18^O Labeled Test Samples

To demonstrate O18Quant's effectiveness, we excised retina samples from a three-month-old male Sprague-Dawley weanling rat (Harlan Inc., Indianapolis, IN) as described previously [19]. The excised retinas were suspended in 400 *μ*L of 100 mM ammonium bicarbonate containing a protease inhibitor cocktail (Sigma-Aldrich, St. Louis, MO), and proteins were extracted by ultrasonication (4.5 kHz three times for 9 s with a 3 min pause on ice between the strokes) using a VirSonic 100 ultrasonic cell disrupter (SP Scientific, Gardiner, NY). The resulting protein extract was centrifuged at 15,000 g for 10 min, and the supernatant was collected. The proteins in the supernatant were then precipitated by mixing with a 4-fold excess volume of ice-cold acetone and left for 1 h at −20°C. The protein precipitate was solubilized in 400 *μ*L of formic acid-methanol (1 : 1, v/v) and subjected to performic acid oxidation to oxidatively cleave disulfide bonds [20]. After the reaction, the reaction mixture was dried in a SpeedVac, redissolved in 200 *μ*L of 100 mM ammonium bicarbonate containing 2 M urea, and the amount of dissolved protein was determined with a DC protein assay kit (Bio-Rad, Hercules, CA). A total of 100 *μ*g of protein was digested by trypsin (1 : 50 substrate to protein ratio, w/w) at 25°C for 16 h. After the digestion, the digest was desalted using Vydac C18 UltraMicro Tip Column (The Nest Group, Southborough, MA) as per the manufacturer's instructions, divided equally into two tubes, and dried in a SpeedVac. Then, the digests were redissolved in 100 *μ*L 100 mM* N*-ethylmorpholine-acetic acid buffer at pH 6 made either with H_2_^16^O or H_2_^18^O. The peptides were then incubated with trypsin (1 : 50 substrate to protein ratio, w/w) at 25°C for 16 h to incorporate ^16^O or ^18^O, respectively, into the carboxyl termini of the peptides [21]. Following the oxygen labeling reaction, the reaction mixtures were dried, redissolved in 100 *μ*L formic acid-methanol (1 : 1, v/v), and subjected to performic acid oxidation to inactivate trypsin.

### 2.2. LC-MS/MS Analysis

The resulting ^16^O and ^18^O labeled samples were dissolved in 0.1% formic acid, mixed in 1 : 2, 1 : 1, and 2 : 1 ratios, and analyzed by LC-MS/MS using an UltiMate 3000 LC system (Dionex, San Francisco, CA, USA) interfaced to an LTQ-Orbitrap XL mass spectrometer (Thermo-Finnigan, Bremen, Germany) [22]. Peptides were chromatographed on a reverse phase column (C18, 75 *μ*m × 150 mm, 3 *μ*m, 100 Ǻ; Dionex) using a linear gradient of acetonitrile from 0% to 40% in aqueous 0.1% formic acid over a period of 90 minutes at 300 nL/minute. The mass spectrometer was operated in a data-dependent MS to MS/MS switching mode, with the eight most intense ions in each MS scan subjected to MS/MS analysis. MS spectra were acquired at 60,000 resolution (FWHM) in the Orbitrap detector (~1 s cycle time) and MS/MS spectra were in the ion trap by collision-induced dissociation (CID). Automatic gain control (AGC) target for MS acquisition was set to 5 × 10^5^. Maximum ion injection times for MS1 and MS2 were 500 and 100 ms, respectively. The threshold intensity for the MS/MS trigger was set at 1,000 and normalized collision energy (NCE) at 35. The data were collected in profile mode for the full scan and in centroid mode for the MS/MS scans. The dynamic exclusion function for previously selected precursor ions was enabled during the analysis such that the following parameters were applied: repeat count of two, repeat duration of 45 s, exclusion duration of 60 s, and exclusion size list of 150. Xcalibur software (version 2.2, Thermo-Finnigan Inc.) was used for instrument control, data acquisition, and data processing.

### 2.3. Mass Spectrometry Data Analysis

Proteins were identified by comparing all of the experimental peptide MS/MS spectra to the Swiss-Prot database using Mascot database search software (version 2.3.2, Matrix Science, London, UK). Oxidation of cysteine to cysteic acid and methionine to methionine sulfone was set as fixed modifications while the modification of the C-terminal carboxyl group with ^18^O was a variable modification. The mass tolerance was set to 10 ppm for the precursor ion and to 0.8 Da for the product ion. Strict trypsin specificity was applied, allowing for one missed cleavage. Only peptides with a minimum score of 20 were considered significant. BuildSummary [23] was used to generate a confident protein list with a false discovery rate for both peptide and protein of ≤0.01. Only the proteins with at least two unique peptides were used in quantification analysis.

### 2.4. Peptide Abundance Estimation

For each identified peptide with observed mass-to-charge* m/z*, charge* z*,^18^O modification state *s*, and user-defined purity of H_2_^18^O  *p*, the abundance of the peptide from the light sample* A* (light) and the heavy sample* A* (heavy) was calculated.

The nature form, the heavy forms with one or two ^18^O labels of the peptide as ^16^O, ^18^O_1_, and ^18^O_2_, and the abundance of those three forms are* A* (^16^O),* A* (^18^O_1_), and* A* (^18^O_2_), the corresponding mass-to-charge of those three forms can be theoretically predicted by formula (1a) to (1c), respectively:

(1a) mz(O16)={mz−2∗dzif  s=modifiedmif  s=unmodified,

(1b) mz(O181)={mz−dzif  s=modifiedmz+dzif  s=unmodified,

(1c) mz(O182)={mzif  s=modifiedmz+2∗dzif  s=unmodified,

where *d* = mass(^18^Oisotope) − mass(^16^Oisotope).

The potential isotope cluster of the peptide is predicted as *mp* = {*m*(^16^O), *m*(^16^O) + *c*, *m*(^18^O_1_), *m*(^18^O_1_) + *c*, *m*(^18^O_2_), *m*(^18^O_2_) + *c*}, where *c* = mass(^13^C  isotope) − mass(^12^C  isotope).

From the scan in which the peptide is identified, the ions of the potential isotope cluster in previous and next scans are extracted from the raw data until the charges of both *m*/*z*(^16^O) and *m*/*z*(^18^O_2_) ions equal zero meaning there is not enough evidence to support the isotope cluster in that scan. Assuming that there are *n* scans containing a potential isotope cluster, an overall observed abundance vector *Y* = {*y*_1_, *y*_2_, *y*_3_, *y*_4_, *y*_5_, *y*_6_} is calculated by formula (2) where *i* indicates the position of the ion in the isotope cluster and *k* indicates the *k*th scan. Consider

(2) $$\begin{array}{c} y_{i}=\overset{n}{\underset{k=1}{\sum }}a_{k,i}. \end{array}$$

A theoretical isotopic abundance vector *V* = {*v*_1_, *v*_2_, *v*_3_, *v*_4_, *v*_5_, *v*_6_} is generated by emass algorithm [24] based on the sequence of the peptide *p*. Then a matrix *X* is constructed, where each row indicates the theoretical isotopic abundance contributed by ^16^O, ^18^O_1_, and ^18^O_2_, respectively, for an ion in the isotope cluster. Consider

(3) $$\begin{array}{c} X=\left[\begin{array}{ccc} v_{1} & 0 & 0\\ v_{2} & 0 & 0\\ v_{3} & v_{1} & 0\\ v_{4} & v_{2} & 0\\ v_{5} & v_{3} & v_{1}\\ v_{6} & v_{4} & v_{2} \end{array}\right]. \end{array}$$

The expected abundance vector *A* = {*A*(^16^O), *A*(^18^O_1_), *A*(^18^O_2_)} can be estimated by solving the formula (4) using the nonnegative least square model:

(4) $$\begin{array}{c} \overset{-}{y}=X*\overset{-}{A}. \end{array}$$

Then,* A* (light) and* A* (heavy) are calculated by the method described by Mason et al. [13].

### 2.5. Protein Quantification

For each protein, multiple peptides may be detected and quantified. Other than combining an outlier rejection scheme with other peptide-to-protein algorithms [25], a robust fitting of linear models is used in our method to estimate the protein ratio from the unlabeled and labeled abundance of each peptide. Detailed information about the algorithm is described in the R package “MASS” (http://cran.r-project.org/web/packages/MASS/index.html).

## 3. Results and Discussion

### 3.1. Implementation

**Figure 1:**
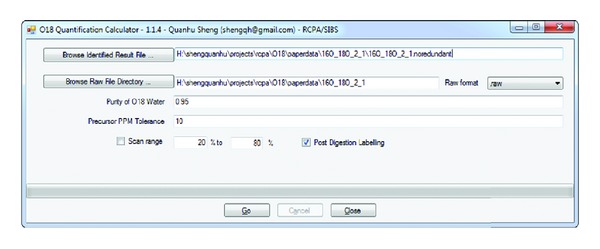
O18 Quantification calculator. The interface is used to calculate peptide/protein relative ratios automatically. User can control the values of various parameters and load in raw data using this interface.

**Figure 2:**
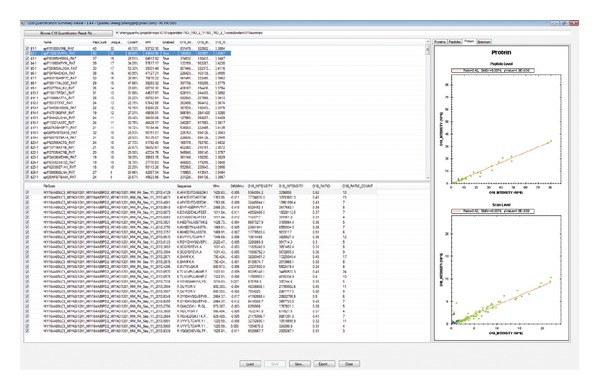
O18 Quantification Summary Viewer. The interface is used to validate the quantification result at protein/peptide/scan level.

The software was implemented using C# and R. R environment is required for peptide-to-protein ratio calculation. Two GUIs are built into O18Quant. The first GUI, O18 Quantification Calculator (Figure 1), is used to automatically extract ions of a potential isotope cluster from the raw file, calculate peptide abundance, estimate protein ratios, and export preliminary quantification result to a tab delimited file. The second GUI, O18 Quantification Summary Viewer (Figure 2), is used to load the preliminary quantification result, validate the result at protein, peptide, and scan levels, and export the validated results. The protein and peptide information are displayed in a spread sheet (Figure 2 left). The scatter plot and RLM fitted line can be visualized within this GUI (Figure 2 right). This GUI allows users to perform visual quality control and manually exclude proteins and peptides with problematic ratios using simple point and click controls.

### 3.2. Visualization and Validation

Three levels of quantification information were stored and visualized for validation.

1. *Protein Level.* Ideally, the peptides from the same protein should have similar relative ratios. From the plot of light/heavy abundances of peptides for each protein, we can easily identify outlier peptides for further validation.
2. *Peptide Level.* For the questionable peptides, the overall scan information of each peptide can be used to validate if the LC peak boundary is properly detected.
3. *Scan Level.* The profile of ion intensity in each scan can be used to validate the scan quality.

To demonstrate the practicality and efficiency of O18Quant's visualization functionality, we chose the protein ATP5I_RAT with the highest ratio of 65.8 in a 2 : 1 sample to validate if that ratio was correct (see Supplementary 1 Figure 1, the first entry in top left table and the red spots in top right and bottom right graphs, in Supplementary Material available online at http://dx.doi.org/10.1155/2014/971857). Two peptides were quantified in protein ATP5I_RAT with respective ratios of 3.09 and 50 (Supplementary Figure 1, the bottom left table). The one with sequence R.YSYLKPR.A and ratio 50 was highly questionable. The corresponding peptide validation page was opened by double clicking the peptide entry (Supplementary 1, Figure 2). The first seven scans contained an unusual 18O(1) ion whose abundance was larger than both 16O and 18O(2) ions. The directions of mass difference between theoretical and observed 16O/O18(1)/O18(2) ions were also different between the first seven scans and the last four scans. Both observations indicate that the detected ions in the first seven scans might belong to another peptide with very similar elution time, and the precursor *m*/*z* of that peptide was very close to the 18O(1) ion of peptide YSYLKPR. Then, the peptide abundance was calculated using only the last four scans. The ratio of the peptide became 3.36 and the ratio of the protein became 3.13, which was more similar to the designed ratio. Detailed validation procedures are described at Supplementary 1.

### 3.3. Quantification Result

Table 1 illustrates the identified and quantified peptides/proteins in three known-ratio samples. All proteins with at least two peptides identified were quantified while some proteins with only one peptide identified failed to be quantified. Here, unique peptides mean peptides with identical sequences without considering their modification states.

**Figure 3:**
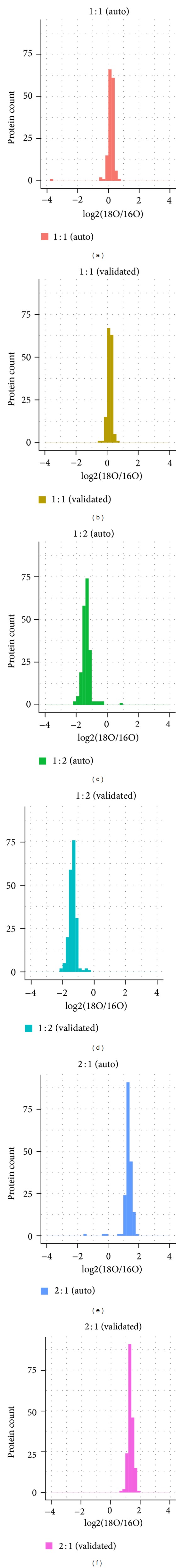
Histogram of log2(ratio) for the three known-ratio samples. Top/bottom three graphs were generated from the data before/after manual validation.

The quantification result before and after careful manual validation of the three samples with designed labeled/unlabeled ratio 1 : 1, 1 : 2, and 2 : 1, respectively, was illustrated as in Figure 3. Only proteins with at least two unique peptides identified were used. The mean and standard deviation of log2(ratio) before manual validation from the three samples were 0.15 ± 0.34, −1.35 ± 0.29, and 1.34 ± 0.47. After careful validation of the peptides with extreme ratios, the mean and standard deviation of log2(ratio) from the three samples became 0.18 ± 0.14, −1.38 ± 0.23, and 1.35 ± 0.17. The standard deviations decreased significantly.

### 3.4. Export Protein/Peptide Summary

After manual validation, the quantification result can be exported to CSV format at protein, peptide, and scan levels for further analysis with additional customizable features. O18Quant allows the protein and peptide level quantification information to be exported into single or separated files. O18Quant is the only tool publicly available now that can export the quantification result at all three levels.

## 4. Conclusions

Proteomic research remains a key component in unlocking the treatment of many human diseases. Here, we present O18Quant, a software package implemented using Microsoft.net framework (C#) and R. O18Quant improves the previous ^18^O/^16^O estimation algorithms in two major areas. First, we employed the RLM model to account for the effect of outliers/extreme values rather than removing them. Second, O18Quant can automate the process of calculating the peptide/protein relative ratio with an intuitive user-friendly GUI. The GUI provides tremendous convenience for users to conduct validation of the quantification results at scan, peptide, and protein levels. O18Quant is free and it will be consistently supported in the coming years.

## Supplementary Material

The protein ATP5I RAT with the highest ratio of 65.8 in a 2:1 sample was chosen to validate if that ratio was correct.
Two peptides were quantified in protein ATP5I RAT with respective ratios of 3.09 and 50. The questionable one with sequence R.YSYLKPR.A and ratio 50 was validated at individual scan level. The peptide and protein ratio became reasonable after manual validation and correction.

## Figures and Tables

**Table 1:** Identified and quantified proteins from three known-ratio samples.

Sample	Identified peptides	Identified proteins	Identified unique 2 proteins*	Quantified peptides	Quantified proteins	Quantified unique 2 proteins*
O18/O16 = 1 : 1	752	257	138	726	251	138
O18/O16 = 1 : 2	993	325	180	961	315	180
O18/O16 = 2 : 1	813	281	162	779	272	162

*Unique 2 protein means that protein was identified with at least two unique peptides.
