# Supplementary material for: O18Quant: A Semiautomatic Strategy for Quantitative Analysis of High-Resolution 16O/18O Labeled Data
Source: Biomed Res Int. 2014 May 11;2014:971857. doi: 10.1155/2014/971857 (PMC4037588; doi:10.1155/2014/971857)
Supplement: Supplementary file 1 — The protein ATP5I RAT with the highest ratio of 65.8 in a 2:1 sample was chosen to validate if that ratio was correct. Two peptides were quantified in protein ATP5I RAT with respective ratios of 3.09 and 50. The questionable one with sequence R.YSYLKPR.A and ratio 50 was validated at individual scan level. The peptide and protein ratio became reasonable after manual validation and correction. [file 971857.f1.pdf]

Supplementary

Figure 1, main interface of O18 Quantification Summary Viewer.

Figure 2, visualization and validation of peptide YSYLKPR.

Figure 3, experimental envelope corresponding to peptide YSYLKPR in scan 2826.

Figure 4, experimental envelope corresponding to peptide YSYLKPR in scan 2833.

Figure 5, experimental envelope corresponding to peptide YSYLKPR in scan 2840.

Figure 6, experimental envelope corresponding to peptide YSYLKPR in scan 2847.

Figure 7, experimental envelope corresponding to peptide YSYLKPR in scan 2854.

Figure 8, experimental envelope corresponding to peptide YSYLKPR in scan 2861.

Figure 9, experimental envelope corresponding to peptide YSYLKPR in scan 2868.

Figure 10, experimental envelope corresponding to peptide YSYLKPR in scan 2875.

Figure 11, experimental envelope corresponding to peptide YSYLKPR in scan 2881.

Figure 12, experimental envelope corresponding to peptide YSYLKPR in scan 2889.

Figure 13, experimental envelope corresponding to peptide YSYLKPR in scan 2896.

Figure 14, optimization of scan range made the O18/O16 ratio of peptide YSYLKPR more accurate.

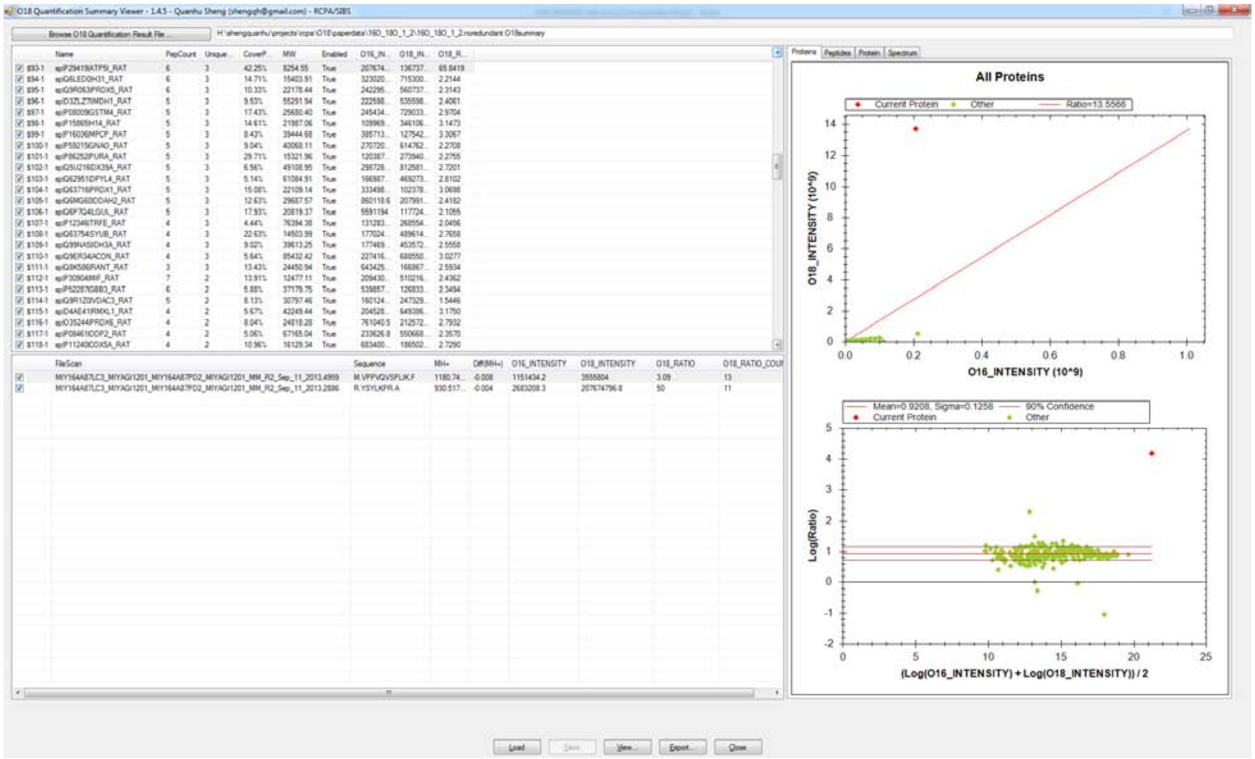

Supplementary Figure 1, O18 quantification summary viewer interface. This figure showed the screen after loading the preliminary result generated by O18 Quantification Calculator. Top left table listed the quantified proteins and bottom left table listed the corresponding quantified peptides of the selected protein. The top right scatter plot and the bottom right MA-plot displayed the abundance of all proteins in unlabeled and labeled sample. The first entry in top left table and the red spots in both top right and bottom right spot indicated the ratio of protein ATP5I_RAT was far away from other proteins. The bottom left table illustrated that there were two peptides with ratio 3.09 and 50 quantified for protein ATP5I_RAT. The peptide R.YSYLKPR.A with ratio 50 was highly questionable. A validation page could be opened by double clicking this entry.

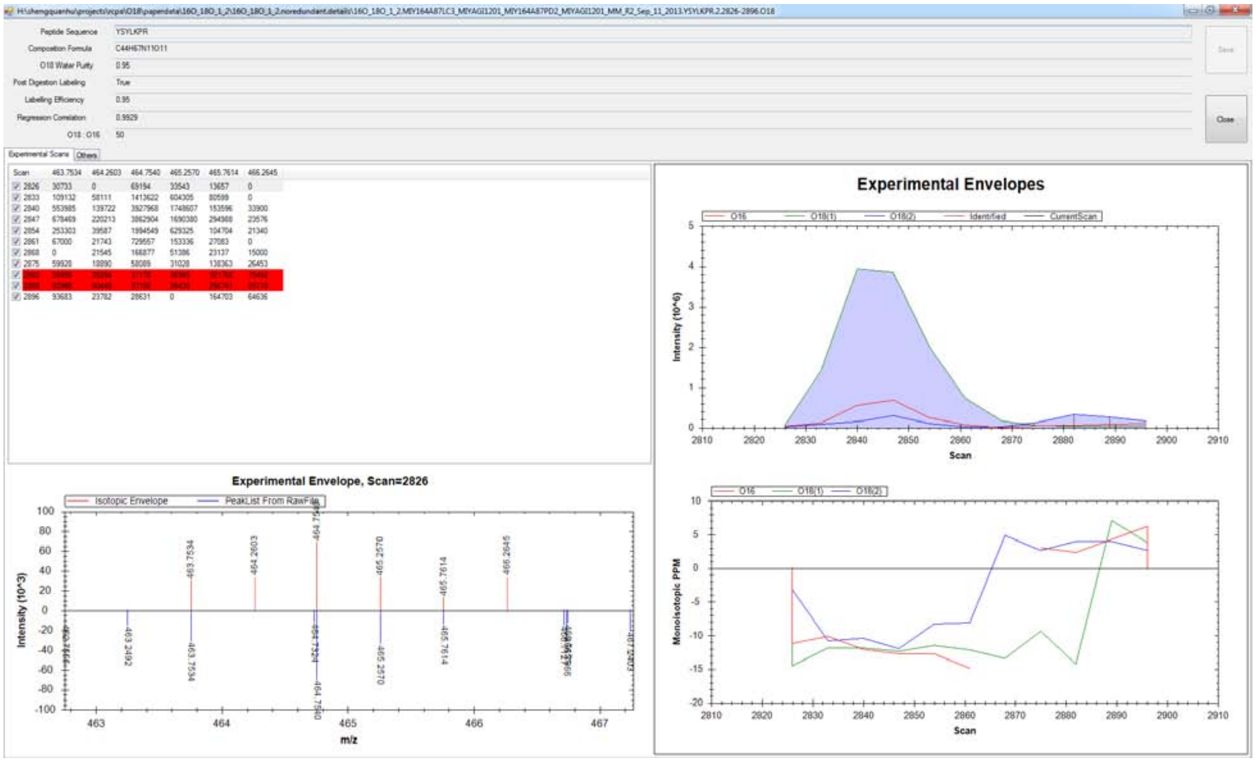

Supplementary Figure 2, Visualization and validation of the peptide YSYLKPR. The middle left table indicated that the first seven scans (from 2826 to 2868) containing unusual $^{18}O(1)$ ion (with m/z 464.7540) whose abundance was higher than both ^{16}O (with m/z 463.7534) and $^{18}O(2)$ ions (with m/z 465.7614). Those seven scans also had different directions of mass difference between theoretical and observed $^{16}O/^{18}O(1)/^{18}O(2)$ ions with the next four scans (bottom right graph, around -10 ppm vs around 5 ppm). Both observations indicated that the detected ions in the first seven scans might belong to another peptide with very similar elution time and the precursor m/z of that peptide was very close to $^{18}O(1)$ ion of the peptide YSYLKPR. Two scans (2882, 2889) marked as red in middle left table indicated that those two scans having tandem mass spectrum identified as the peptide YSYLKPR.

Supplementary Figure 3-13 show the detailed ion information of each scan. By visualization, the last four scans (from 2875-2896) were used in peptide ratio calculation (Supplementary Figure 14). Based on the last 4 scans, the peptide YSYLKPR had ratio 3.36 and the protein ATP5I_RAT had ratio 3.13 which were more similar to the designed ratio.

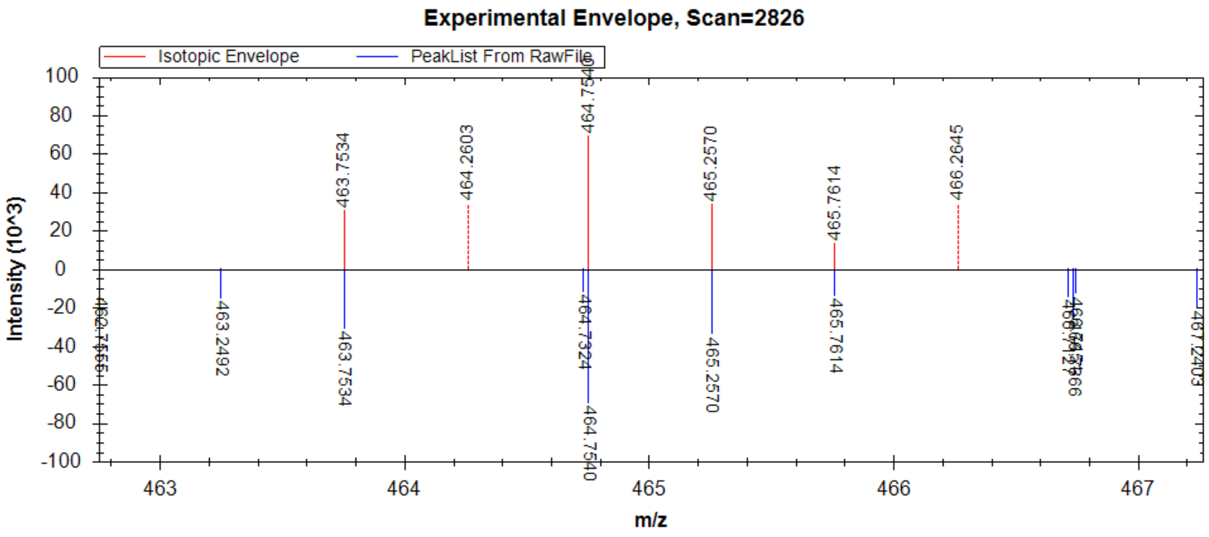

Figure 3, experimental envelope of scan 2826. The red solid line means that the theoretical ion was observed at that scan. The red dot line means that the theoretical ion was not observed at that scan. The length of solid line indicated the abundance of that ion.

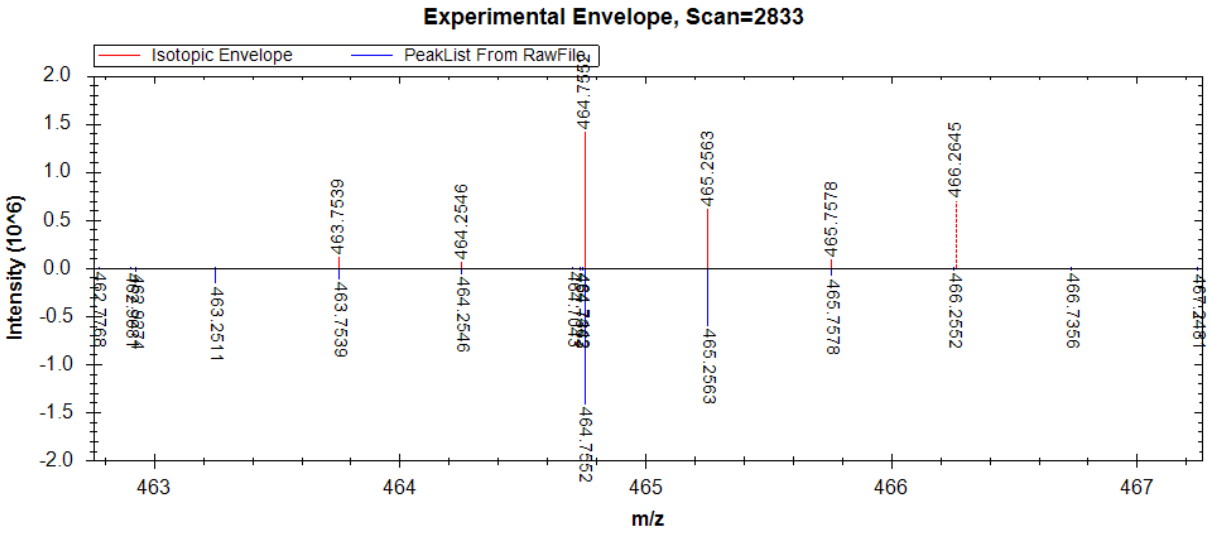

Figure 4, experimental envelope of scan 2833.

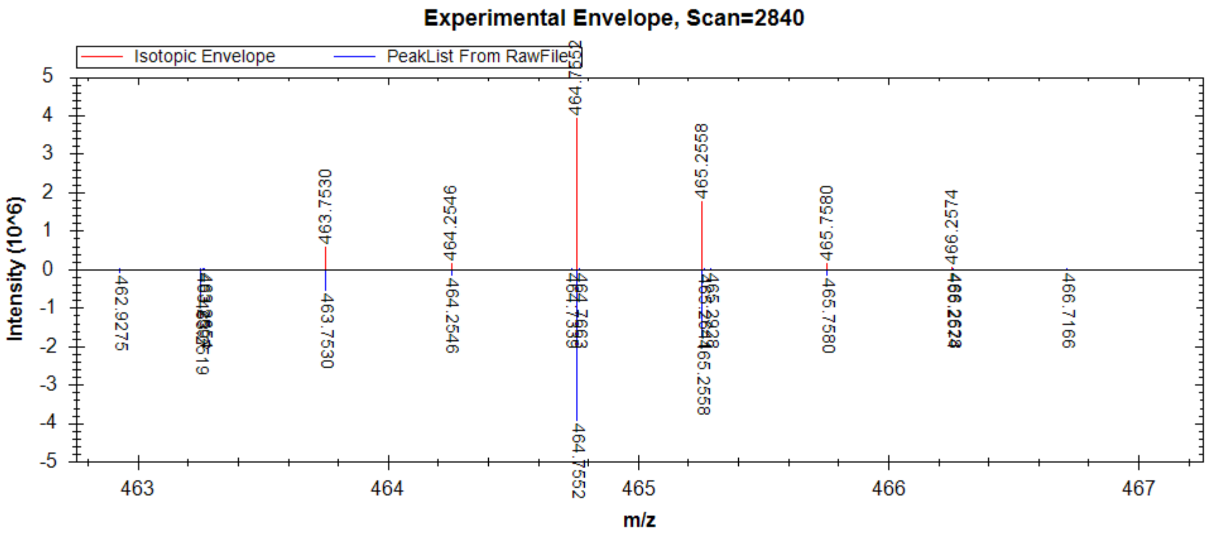

Figure 5, experimental envelope of scan 2840.

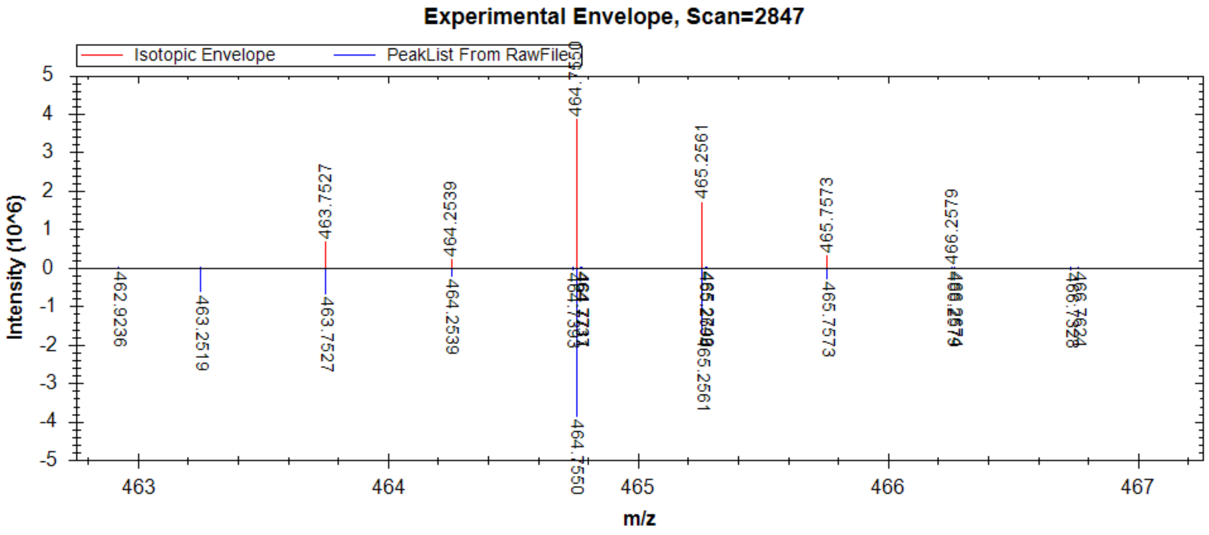

Figure 6, experimental envelope of scan 2847.

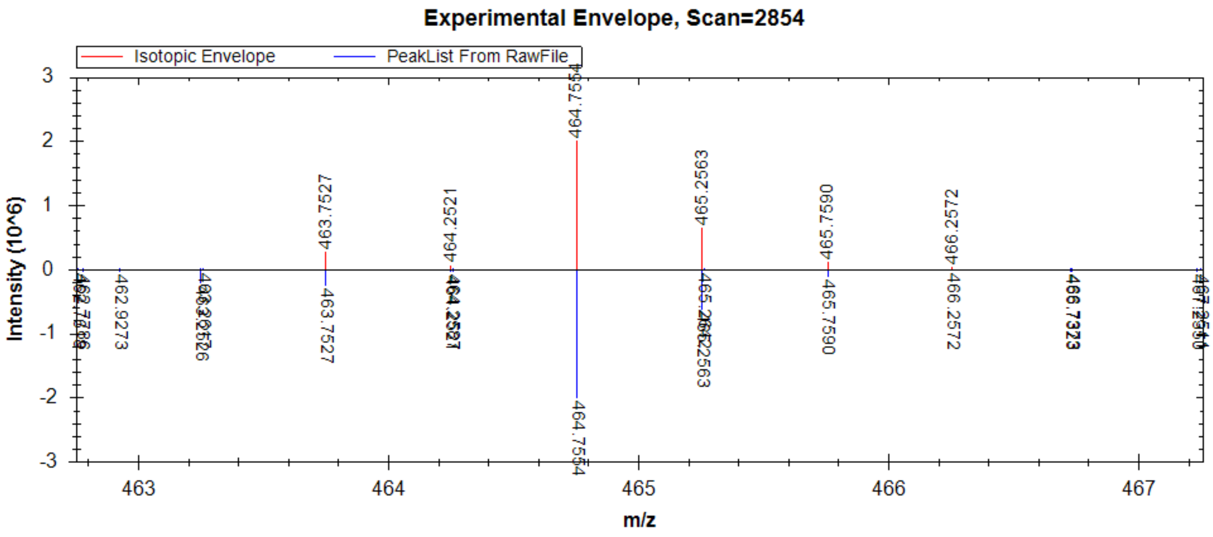

Figure 7, experimental envelope of scan 2854.

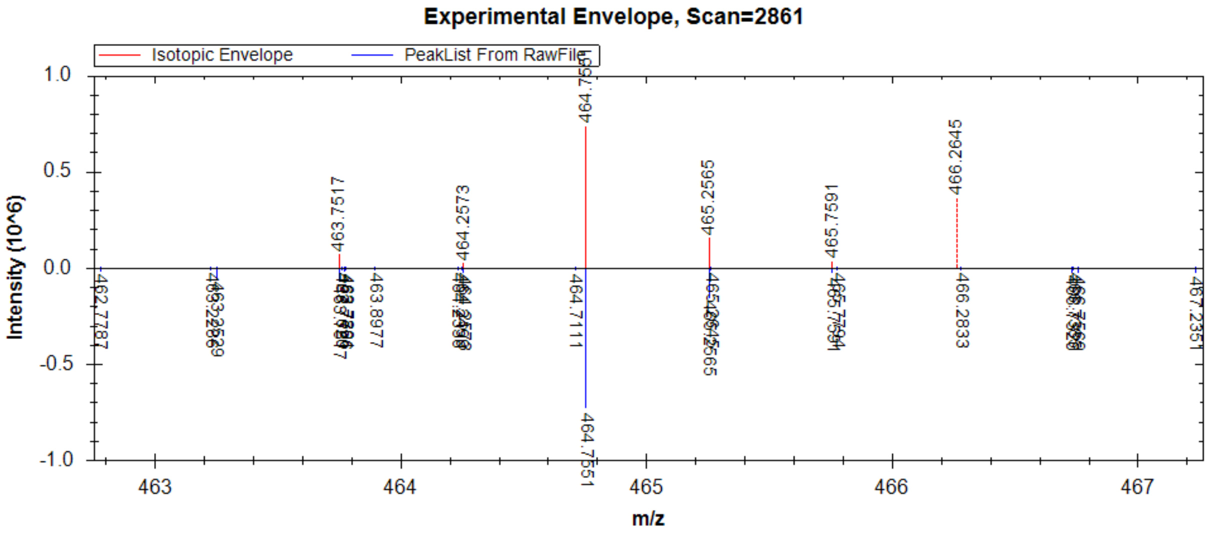

Figure 8, experimental envelope of scan 2861.

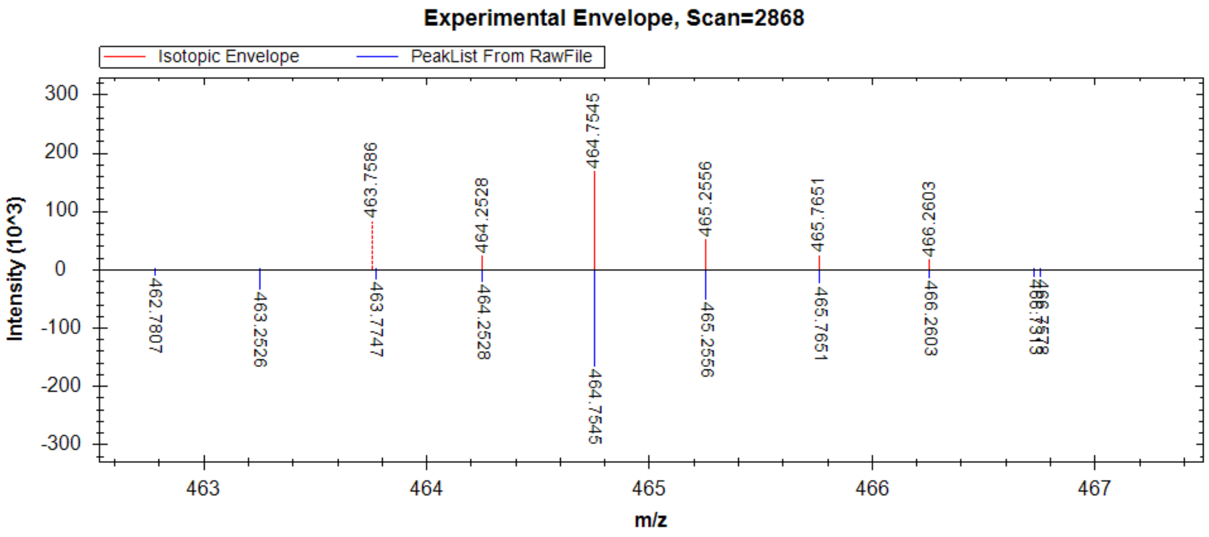

Figure 9, experimental envelope of scan 2868.

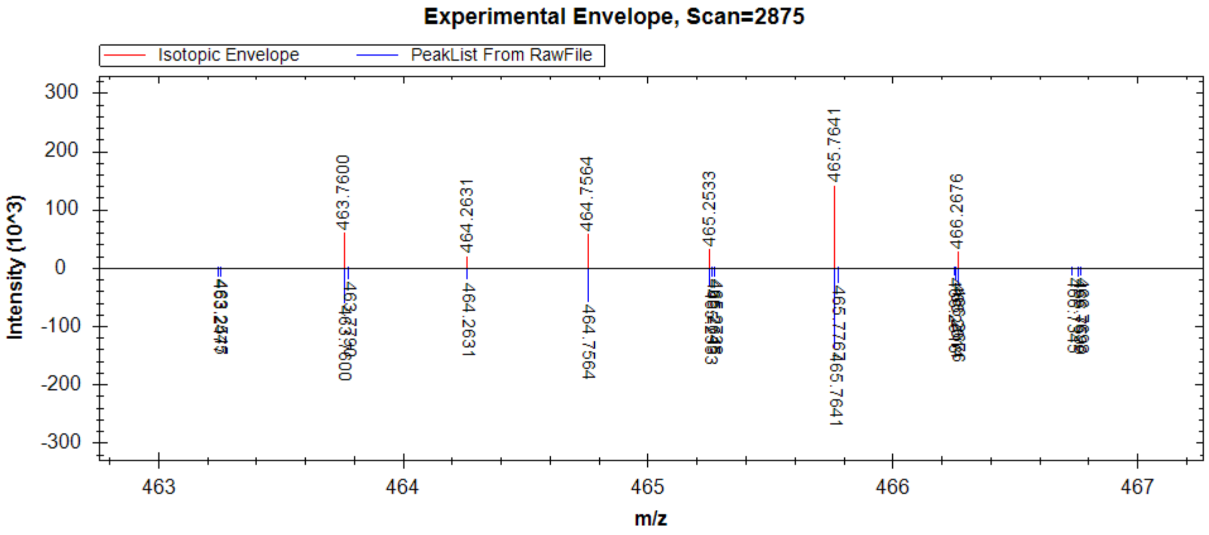

Figure 10, experimental envelope of scan 2875.

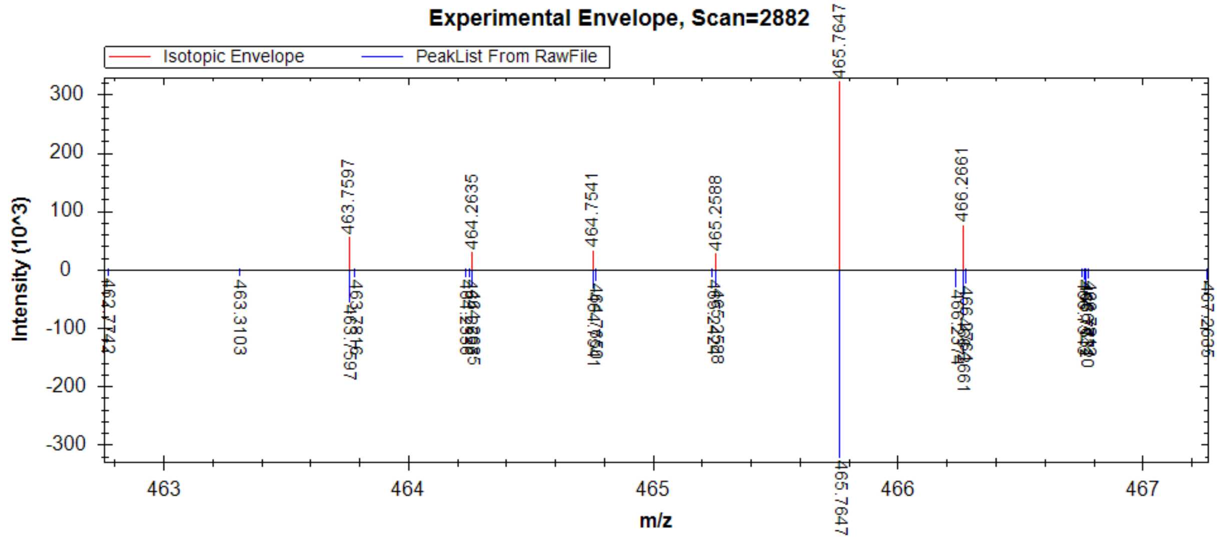

Figure 11, experimental envelope of scan 2882.

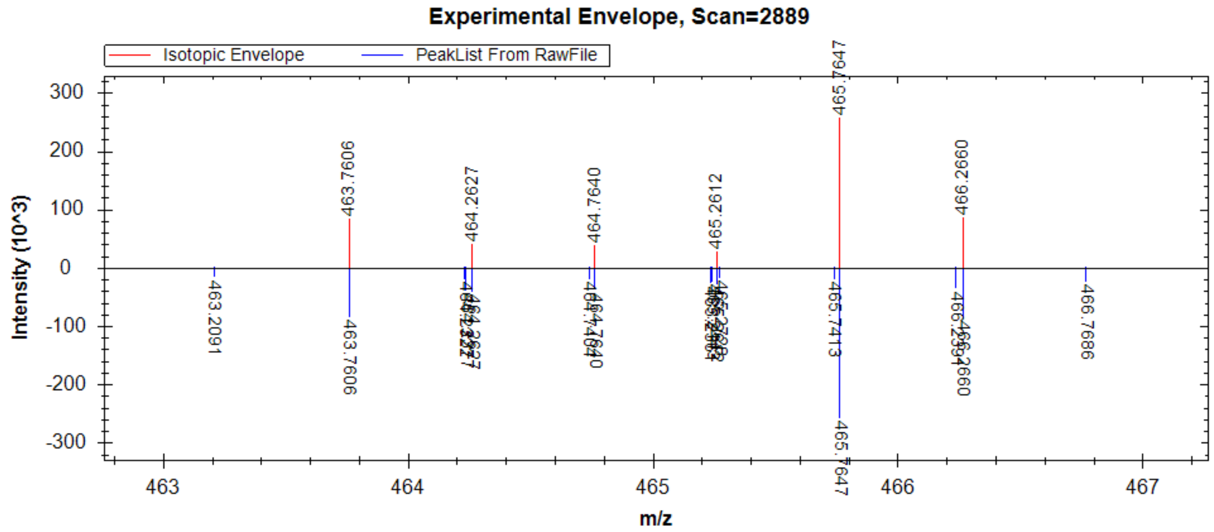

Figure 12, experimental envelope of scan 2889.

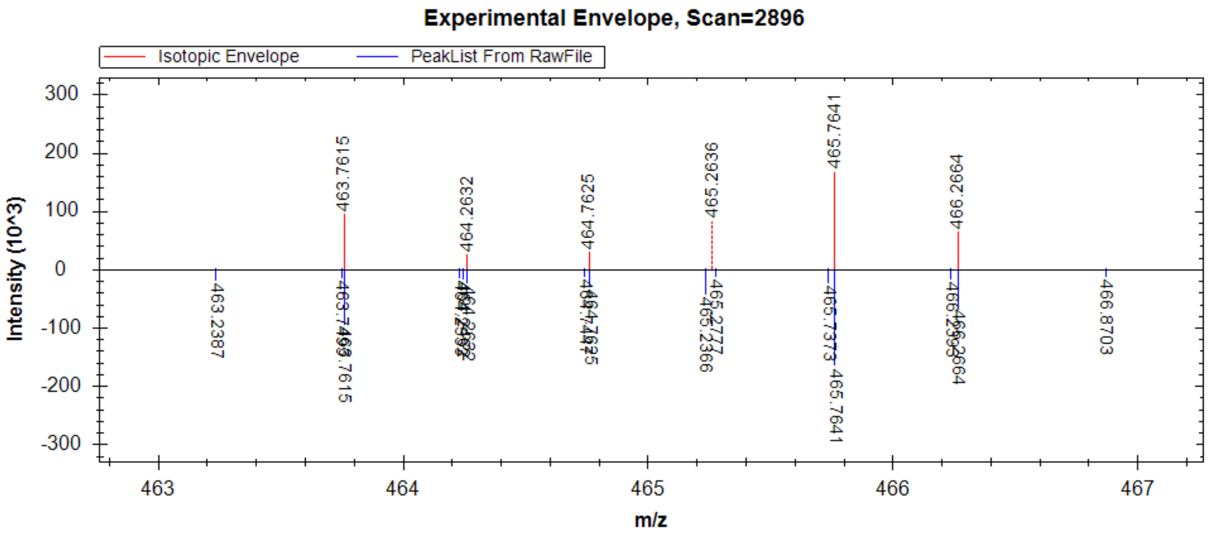

Figure 13, experimental envelope of scan 2896.

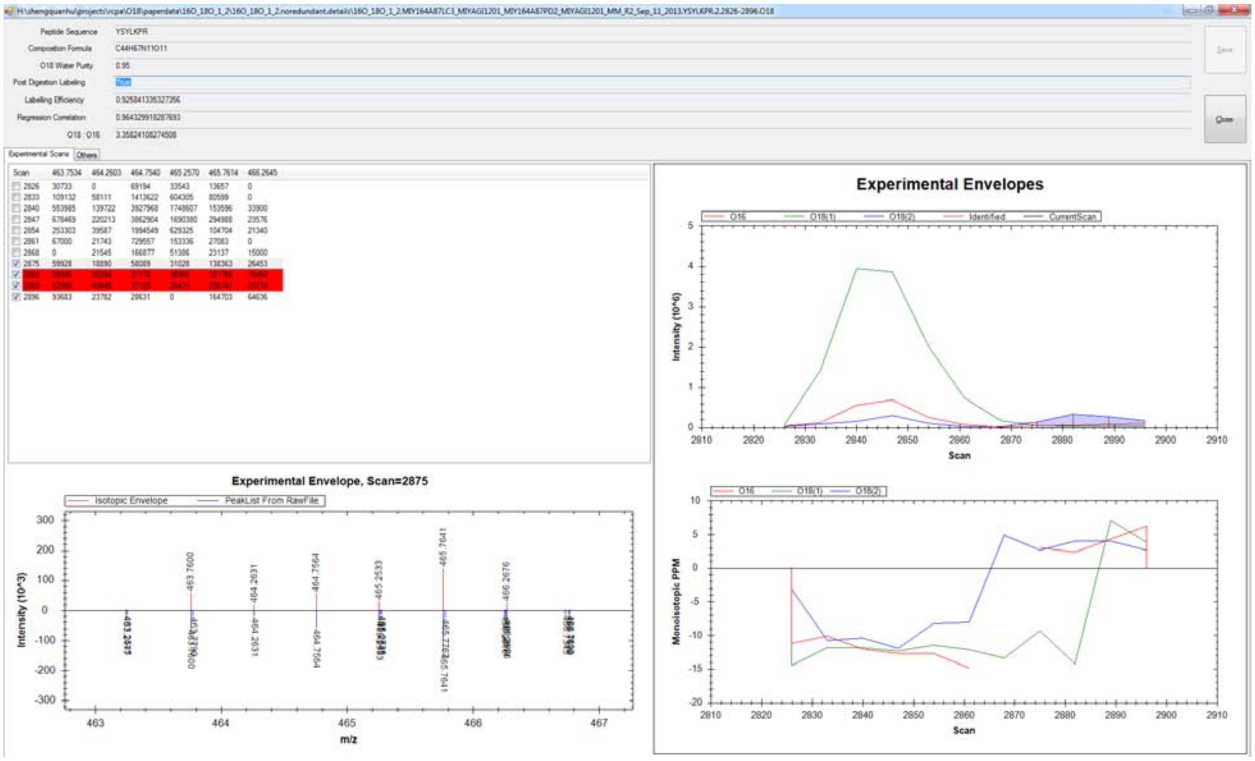

Figure 14, optimization of scan range made the O18/O16 ratio of peptide YSYLKPR more accurate.
